# The impact of diabetes mellitus on acute kidney injury after coronary artery bypass grafting

**DOI:** 10.1186/s13019-020-01312-x

**Published:** 2020-10-01

**Authors:** Rui Wang, Hang Zhang, Yifan Zhu, Wen Chen, Xin Chen

**Affiliations:** Department of Cardiovascular Surgery, Nanjing First Hospital, Nanjing Medical University, Nanjing, 68 Changle Rd, Nanjing, 210006 People’s Republic of China

**Keywords:** Diabetes mellitus, Coronary artery bypass grafting, Acute kidney injury, Insulin

## Abstract

**Background:**

Diabetes mellitus(DM) is an indicator affects postoperative mortality and morbidity after coronary artery bypass grafting (CABG). Acute kidney injury (AKI) is one of the frequent postoperative complications after CABG. This multi-centre register study designed to investigate the impact of DM on postoperative AKI in primary isolated CABG patients.

**Methods:**

We included all patients (*n* = 4325) from Jiangsu Province CABG register who underwent primary isolated CABG from September 2017 to August 2019. The patients were divided into 3 groups: No-DM group(*n* = 3067), DM-oral group (DM with oral hypoglycemic agents, *n* = 706) and DM-insulin group (DM with insulin treatment, *n* = 552). The development and severity of AKI were based on Acute Kidney Injury Network (AKIN) criteria.

**Results:**

There were totally 338, 108 and 145 patients developed AKI in No-DM, DM-oral and DM-insulin group respectively. Comparing with No-DM group, DM-oral group had a higher rate of AKI(χ^2^ = 10.071, *p* = 0.002), DM-insulin group had a higher rate(χ^2^ = 94.042, p<0.001) and severity of AKI(χ^2^ = 10.649, *p* = 0.005). The adjusted odds ratio for AKI was 1.26 (95% CI 1.03–1.57) in DM-oral group and 3.92 (95% CI 3.27–5.16) in DM-insulin group, in comparison with No-DM group.

**Conclusions:**

Independent of baseline renal function or cardiac function, DM was associated with an increased risk of AKI after CABG, especially in patients with insulin treatment, who also had a higher severity of AKI.

## Background

Coronary artery bypass grafting (CABG) is recognized as one of the most effective procedures for the treatment of coronary artery atherosclerosis disease. Among patients undergoing CABG, diabetes mellitus (DM) accounted for 20–50% [1–3], and the proportion has increased steadily over the last 15 years [4, 5].

DM is a known risk factor for developing postoperative acute kidney injury (AKI) [6, 7]. AKI is a sudden loss of kidney function defined by an acute increase in serum creatinine concentration and decrease in urinary output [8]. Up to 30% of patients with varying severity develop AKI after cardiac surgery, and approximately 2% require temporary dialysis [9]. Postoperative AKI is associated with increased short and long term morbidity and mortality [10, 11]. Furthermore, DM is one of the major causes of nephropathy following CABG surgery [12]. However, there are limited studies which specifically investigated the different risk of AKI between patients with oral hypoglycemic treatment and insulin treatment after CABG. The purpose of this study was to investigate the association between DM and AKI following primary isolate CABG, and to explore the effect of different treatment of DM on AKI.

## Methods

### Definition of renal function

The study was an observational province wide cohort study which included 13 cardiac centres in Jiangsu province. Data were collected routinely as part of a province CABG register, the register website is: http://221.226.218.114:10004/Multicenter. The Province-wide CABG Registry system was set in Nanjing First Hospital, the study was approved by the Ethics Committee of Nanjing First Hospital, and patient’s identifiers were removed before analysis. DM was defined as the requirement for dietary modification, oral agents and/or insulin to lower blood glucose concentrations and was accepted as present based on the patient’s history corroborated where possible by the medical records. The estimated glomerular filtration rate (eGFR) was calculated by the abbreviated Modification of Diet in Renal Disease equation:186 × (serum creatinine/88.4)^-1.154^ × (age)^-0.203^ × (0.742 if female). AKI was defined and classified according to the criteria proposed by the Acute Kidney Injury Network (AKIN) as AKIN stage 1: increase creatinine × 1.5 from baseline or increase of > 0.3 mg/dL within 48 h; AKIN stage 2: increase creatinine × 2 from baseline; and AKIN stage 3: increase in creatinine × 3 from baseline or creatinine > 4 mg/dL with an acute increase > 0.5 mg/dL within 48 h or new-onset of dialysis therapy [13].

### Study population

A standard set of perioperative data was collected prospectively for patients undergoing primary isolated CABG who were enrolled in Jiangsu province CABG register between January 2017 and December 2019.

Patients undergoing a concomitant cardiac surgical procedure, reoperation, urgent or emergent operations, or with incomplete information were excluded. Totally there were 4325 cases up to the standard which were divided into 3 groups: No-DM group(*n* = 3067, 70.9%)), DM-oral group(DM with oral hypoglycemic agents, *n* = 706, 16.3%) and DM-insulin group(DM with insulin treatment, and with or without oral hypoglycemic agents, *n* = 552, 12.8%). On-pump CABG was performed via median sternotomy using a membrane oxygenator equipped with an arterial filter, cold blood antegrade cardioplegia under moderate systemic hypothermia (30 to 34 °C). The perfusion pressure during cardiopulmonary bypass(CPB) was maintained within 60–70 mmHg. Off-pump CABG was performed by a suction stabilizer. Good exposure of lateral vessels might be got by using deep pericardial retraction sutures. Visualization was enhanced by using a blower device. Intra-coronary shunt was used routinely. Average of perioperative blood sugar level was tried to be controlled under 150 mg/dl by oral hypoglycemic agents and/or insulin treatment.

Twenty-one perioperative variables were collected including: age, gender, body mass index(BMI), eGFR, smoking, hypertension, DM and the type of treatment, hyperlipemia, chronic obstructive pulmonary disease(COPD), peripheral vascular disease(PVD), prior cerebro-vascular accident(CVA), myocardial infarction(MI) and percutaneous coronary intervention(PCI), left ventricular ejection fraction(LVEF), number of vessel disease, EuroSCOREII, number of distal anastomosis, the application of left internal mammary artery(LIMA) and radial artery, application of CPB or not and CPB time, the incidence and severity of AKI.

### Statistical analysis

Data are represented as the mean ± standard deviation unless otherwise indicated. Categorical variables are represented as frequency distributions and single percentages. Normally distributed continuous variables were compared using a Student t-test, non-normally distributed continuous variables using the Mann-Whitney U test, and categorical variables were compared by χ2 test.

The multivariable analysis was adjusted for age, gender, BMI, eGFR, hypertension, hyperlipemia, COPD, PVD, prior CVA, MI and PCI, LVEF, number of vessel disease, EuroSCOREII, number and type of grafts, CPB and CPB time.

All statistical tests were two-sided. A *p*-value of less than 0.05 was considered significant. All statistical analysis were done with IBM SPSS Statistics 20.0 or STATA Data analysis and statistical software.

## Results

### Patient demographics and operative characteristics

The baseline clinical characteristics of the study groups are given in Table 1. Comparing with No-DM group, DM-oral group had a higher percentage of female, higher BMI, higher incidence of PVD, prior MI and PCI, higher EuroSCOREII, higher CPB time, but a lower eGFR. Comparing with No-DM group, DM-insulin group had a higher age, higher percentage of female, higher BMI, higher incidence of hypertension, hyperlipemia and PVD, higher incidence of prior CVA, MI and PCI, higher number of vessel diseases and left main diseases, higher EuroSCOREII, a higher ratio of on-pump CABG and longer CPB time, but a lower eGFR and LVEF, and a lower application of LIMA (Table 1).

**Table 1:** Baseline and procedural characteristics in relation to type of treatment of DM

Variable	All cases	No-DM group	DM-oral group	*p*	DM-insulin group	*p*
	(*n* = 4325)	(*n* = 3067)	(*n* = 706)		(*n* = 552)	
Age, y	64.1 ± 8.4	64.2 ± 8.1	64.8 ± 7.6	0.073	62.7 ± 9.3	< 0.001
Female gender	793(18.3)	506(16.5)	148(21.0)	0.005	139(25.2)	< 0.001
BMI, kg/m^2^	26.7 ± 4.9	26.5 ± 4.6	27.3 ± 5.2	< 0.001	27.1 ± 5.7	0.002
eGFR(ml/min/1.73m^2^)	78.3 ± 18.0	80.3 ± 17.8	76.3 ± 16.3	< 0.001	69.5 ± 19.4	< 0.001
Hypertension	2047(47.3)	1414(46.1)	343(48.6)	0.234	290(52.5)	0.005
Hyperlipemia	1095(25.3)	770(25.1)	161(22.8)	0.201	164(29.7)	0.023
COPD	312(7.2)	221(7.2)	53(7.5)	0.781	38(6.9)	0.787
Peripheral vascular disease	369(8.5)	230(7.5)	71(10.1)	0.024	68(12.3)	< 0.001
Prior						
CVA	253(5.8)	160(5.2)	45(6.4)	0.221	48(8.7)	< 0.001
MI	697(16.1)	463(15.1)	130(18.4)	0.029	104(18.8)	0.026
PCI	739(17.1)	482(15.7)	134(19.0)	0.034	123(22.3)	< 0.001
LVEF				0.867		0.03
> 0.50	3140(72.6)	2248(73.3)	513(72.7)		379(68.6)	
0.30–0.50	1066(24.6)	742(24.2)	173(24.5)		151(27.4)	
< 0.30	119(2.8)	77(2.5)	20(2.8)		22(4.0)	
No. of vessel disease				0.156		0.02
1 vessel	222(5.1)	181(5.9)	29(4.1)		12(2.2)	
2 vessel	473(10.9)	328(10.7)	81(11.5)		64(11.6)	
3 vessel	3630(83.9)	2558(83.4)	596(84.4)		476(86.2)	
Left main disease	1073(24.8)	721(23.5)	179(25.4)	0.299	173(31.3)	< 0.001
EuroSCOREII	2.1 ± 0.8	1.9 ± 0.7	2.4 ± 0.9	< 0.001	3.1 ± 1.1	< 0.001
Distal anastomosis						
LIMA	3954(91.4)	2828(92.2)	637(90.2)	0.083	489(88.6)	0.005
Radial artery	151(3.5)	107(3.5)	26(3.7)	0.801	18(3.3)	0.787
On-pump	1854(42.9)	1270(41.4)	315(44.6)	0.119	269(48.7)	0.001
CPB time(min)	74.2 ± 19.5	71.5 ± 18.9	78.5 ± 20.7	< 0.001	83.5 ± 23.2	< 0.001

No-DM group: non-DM; DM-oral group: DM with oral hypoglycemic agents; DM-insulin group: DM with insulin treatment, and with or without oral hypoglycemic agents. *BMI* body mass index, *eGFR* estimated glomerular filtration rate, *COPD* chronic obstructive pulmonary disease, *CVA* cerebro-vascular accident, *MI* myocardial infarction, *PCI* percutaneous coronary intervention, *LVEF* left ventricular ejection fraction, *LIMA* left internal mammary artery, *CPB* cardiopulmonary bypass

### Risk of AKI in relation to DM

There were totally 338(11.0%), 108(15.3%) and 145(26.3%) patients developed AKI postoperatively in No-DM, DM-oral and DM-insulin group respectively (No-DM group vs. DM-oral group, χ^2^ = 10.071, *p* = 0.002; No-DM group vs. DM-insulin group, χ^2^ = 94.042, p<0.001). After adjustment for confounders, comparing with No-DM group, the risk of AKI in DM-insulin group was close to 4-fold (OR 3.92, 95% CI 3.27–5.16); the risk of AKI in DM-oral group was 1.26, smaller but still significant (OR 1.26, 95% CI, 1.03–1.57). (Table 2).

**Table 2:** Odds ratios with 95% CIs for AKI after CABG in 3 groups

No. of patients	All	No-DM group	DM-oral group	DM-insulin group
	4325	3067	706	552
No. of AKI(%)	591(13.7)	338(11.0)	108(15.3)	145(26.3)
Risk of AKI		OR (95% CI)		
		1	1.46	2.88
(crude analysis)			1.15–1.84	2.31–3.59
Risk of AKI		1	1.26	3.92
(multivariable adjusted^a^)			1.03–1.57	3.27–5.16

AKI was defined as increase creatinine ×1.5 from baseline or increase of > 0.3 mg/dL within 48 h

^a^ The final multivariable model included all variables in Table 1 except EuroSCOREII

### Risk of AKI according to DM after stratified by preoperative renal or cardiac function

When patients were stratified according to eGFR (> 60, 45–60, 15-45 mL/min/1.73 m^2^) or LVEF(> 0.50, 0.30–0.50, < 0.30), the associations between subtypes of different treatment of DM and AKI were similar for patients with reduced eGFR and normal eGFR, or with reduced LVEF and normal LVEF (Tables 3 and 4).

**Table 3:** Risk of AKI after CABG according to oral hypoglycemic and insulin treatment, stratified by preoperative renal function

	All	No-DM group	DM-oral group	DM-insulin group
eGFR ≥60 mL/min/1.73 m^2^				
No. of patients	3456	2484	558	414
No. of AKI(%)	358(10.4)	205(8.3)	63(11.3)	90(21.7)
Risk of AKI		OR (95% CI)		
		1	1.69	3.7
(crude analysis)			1.25–2.28	2.81–4.86
Risk of AKI		1	1.2	4.35
(multivariable adjusted^a^)			0.89–1.66	2.79–6.38
eGFR 45–60 mL/min/1.73 m^2^				
No. of patients	644	461	89	94
No. of AKI(%)	150(23.3)	96(20.8)	23(25.8)	31(32.8)
Risk of AKI		OR (95% CI)		
		1	1.33	1.87
(crude analysis)			0.78–2.24	1.15–3.04
Risk of AKI		1	1.18	2.9
(multivariable adjusted^a^)			0.64–2.07	2.17–5.31
eGFR 15–45 mL/min/1.73 m^2^				
No. of patients	225	122	59	44
No. of AKI(%)	83(36.9)	37(30.3)	22(37.3)	24(54.5)
Risk of AKI		OR (95% CI)		
		1	1.37	2.75
(crude analysis)			0.71–2.63	1.36–5.60
Risk of AKI		1	1.18	4.02
(multivariable adjusted^a^)			0.59–2.17	2.44–6.59

AKI was defined as increase creatinine ×1.5 from baseline or increase of > 0.3 mg/dL within 48 h

^a^ The final multivariable model included all variables in Table 1 except EuroSCOREII

**Table 4:** Risk of AKI after CABG according to oral hypoglycemic and insulin treatment, stratified by preoperative cardiac function

	All	No-DM group	DM-oral group	DM-insulin group
LVEF> 0.50				
No. of patients	3140	2248	513	379
No. of AKI(%)	350(11.1)	203(9.0)	63(12.3)	84(22.2)
Risk of AKI		OR (95% CI)		
		1	1.41	2.87
(crude analysis)			1.04–1.91	2.16–3.80
Risk of AKI		1	1.09	4.11
(multivariable adjusted^a^)			0.79–1.31	2.97–5.05
LVEF 0.30–0.50				
No. of patients	1066	742	173	151
No. of AKI(%)	201(18.9)	114(15.4)	38(22.0)	49(32.5)
Risk of AKI		OR (95% CI)		
		1	1.55	2.65
(crude analysis)			1.03–2.34	1.78–3.93
Risk of AKI		1	1.18	3.47
(multivariable adjusted^⁎^)			0.67–1.84	2.11–4.80
LVEF< 0.30				
No. of patients	119	77	20	22
No. of AKI(%)	40(33.6)	21(27.3)	7(35.0)	12(54.5)
Risk of AKI		OR (95% CI)		
		1	1.44	3.2
(crude analysis)			0.50–4.09	1.20–8.51
Risk of AKI		1	1.15	4.06
(multivariable adjusted^a^)			0.34–3.57	2.19–9.83

AKI was defined as increase creatinine ×1.5 from baseline or increase of > 0.3 mg/dL within 48 h

^a^ The final multivariable model included all variables in Table 1 except EuroSCOREII

### Severity of AKI in relation to DM

**Fig. 1:**
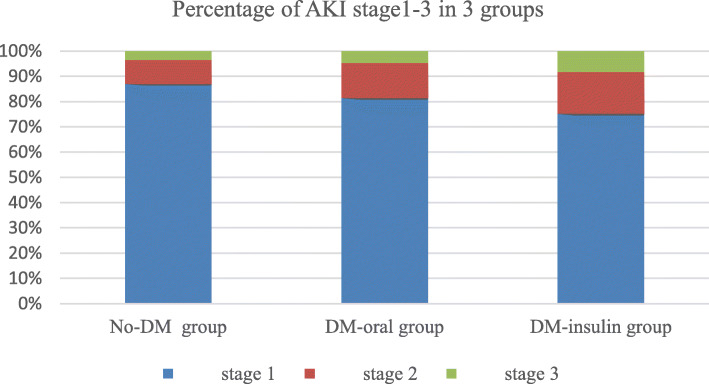
Acute kidney injury (AKI), as defined and classified according to the criteria proposed by the Acute Kidney Injury Network (AKIN), is shown stratified according to kidney function at baseline (blue = AKI stage 1, red = AKI stage 2, green = AKI stage 3; y-axis = percentage of patients)

Comparing with No-DM group, DM-oral group had a higher rate of AKI(108 vs. 338, χ^2^ = 10.071, *p* = 0.002) but the severity of AKI was of no significance(AKIN stage1: 88 vs 294; AKIN stage2: 15 vs 32; AKIN stage3: 5 vs 12, χ^2^ = 2.058, *p* = 0.357). Comparing with No-DM group, DM-insulin group had a higher rate(145 vs. 338, χ^2^ = 94.042, p = 0.002) and severity of AKI(AKIN stage1: 109 vs 294; AKIN stage2: 24 vs 32; AKIN stage3: 12 vs 12, χ^2^ = 10.649, *p* = 0.005) (Table 5 and Fig. 1).

**Table 5:** Rate and severity of AKI after CABG in 3 groups

	No-DM group	DM-oral group	DM-insulin group
All AKI	338	108	145
Stage 1	294(87.0)	88(81.5)	109(75.2)
Stage 2	32(9.5)	15(13.9)	24(16.5)
Stage 3	12(3.5)	5(4.6)	12(8.3)

AKI and classification were defined according to Acute Kidney Injury Network (AKIN)

## Discussion

The principal findings of this province wide CABG register study can be summarized as follows: (1) Comparing with non-DM patients, DM patients were associated with an increased risk of AKI after CABG and were independent of baseline renal function or cardiac function. (2) Among DM patients, patients with insulin treatment were associated with an increased rate and severity of AKI compared with those with oral hypoglycemic agents treatment.

DM is the leading cause of chronic kidney disease(CKD), diabetic nephropathy and is increasing as a cause of morbility and mortality in the present era. Among all these complications, diabetic nephropathy has become the principal cause of end-stage renal failure and cardiovascular mortality [14, 15].

AKI is a frequent complication after cardiac surgery, which is known to have an adverse influence on the patients’ outcomes, including progression to CKD, cardiovascular effects, sustained functional impairment and death. Minimal changes in postoperative creatinine can be associated with adverse effects. This diagnostic criteria for AKI is designed to facilitate the acquisition of a new concept in that small alterations in kidney function might contribute to adverse outcomes. In recent 10 years, over 80 cohort studies including more than 2 million participants have described relationships between AKI and the risks of CKD, diabetic nephropathy and death [16]. However, a limited number of studies have focused on the difference between the type of treatment of DM patients undergoing CABG.

DM is speculated to aggravate AKI by the following complex mechanisms: (1) Hyperglycaemia can increase oxidative stress and amplify ischaemia-reperfusion injury [17]. (2) Celluar glucose overload induces mitochondrial dysfunction and kidney injury [18]. (3) Inflammation is an important factor for the development of kidney injury, and hyperglycaemia is reported to increase inflammatory cytokines such as interleukin-6, tumour necrosis factor-α and interleukin-18 [19]. (4) Endothelial dysfunction induced by hyperglycaemia leads to kidney injury [20]. In this study, DM patients undergoing CABG were associated with an increased risk of AKI compared with non-DM patients. The same result was obtained by Hertzberg D et al., they reported both type 1 and 2 DM were associated with an increased risk of AKI after CABG [21]. A Tekeli Kunt et al. reported the presence of metabolic syndrome (hyperglycemia, dyslipidemia, abdominal obesity, and hypertension) seemed to be associated with increased incidence of AKI after CABG [22]. Oezkur et al. concluded that chronic hyperglycemia defined on a single measurement of hemoglobin A1c ≥ 6.0% was also associated with the incidence of AKI after CABG. All of the above proved that DM was an independent risk factor for AKI after CABG [23].

Our subgroup analyses according to the type of DM treatment showed that when compared with those without DM, the risk of AKI were significantly higher in patients treated with oral hypoglycemic agents, and the risk and severity of AKI were both further significantly increased in patients treated with insulin. The conclusion from Hertzberg D et al. was in line with our study [21]. We speculated the reasons were as follows: (1) Hyperglycaemia, regardless of the presence of DM, is one of the major risk factors associated with poor prognosis including renal dysfunction, and more severe hyperglycaemia were more likely to be treated with insulin. (2) There must be some type 1 DM patients in DM-insulin group, despite we did not classify the type of DM, and type 1 DM was reported to be associated with a significant increased rate and severity of AKI compared with type 2 DM [21].

Preoperative preexisting kidney disease and reduced left ventricular function are well known as important risk factors of postoperative AKI after CABG [24, 25]. For patients with preexisting kidney disease, it could be explained not only by an increased renal vulnerability but also by serum creatinine kinetics whereby an absolute increasement in serum creatinine levels by 0.3 mg/dL is easier to reach when the baseline serum creatinine value is already enhanced. For patients with preexisting reduced left ventricular function, it could be explained not only by global hypoperfusion and renal malperfusion but also by cardiotonics and vasoconstrictors which might damage the renal function. So our study was further stratified by this two risk factors, which were measured in terms of eGFR and LVEF. After stratification, there was still a remakable increasement of postoperative AKI in DM patients compared with non-DM patients, in other words, the association between DM and the risk of AKI was similar in different eGFR or LVEF categories, although few studies have specifically studied this association. Meanwhile, this conclusion was also consistent with our subgroup analyses as mentioned before. Unfortunately, there are no pharmacologic agents known to reduce the risk of AKI or treat established AKI [26]. Therefore, DM patients undergoing CABG need to strengthen the perioperative glucose management and the follow-up of endocrinology.

## Limitations

Firstly, a retrospective, non-randomized register study over a long period of time and with different surgeon’s procedures on patients undergoing CABG is subjected to the effects of selection bias. Secondly, we only classified the DM according to the treatment instead of type(type1 and type2), and we were lack of details of glucose management and levels of hemoglobin A1c. Thirdly, it was impossible for us to stratify all risk factors, such as age, CPB, PVD and so on. Finally, We did not have information on the administration of angiotensin-converting enzyme inhibitors, angiotensin receptor blockers or aldosterone antagonists perioperatively, which could also be related to AKI.

## Conclusions

In summary, this analysis revealed that compared with non-DM patients, DM patients were associated with an increased risk of AKI after CABG irrespective of baseline renal function or cardiac function. The rate and severity of AKI were remarkable higher in DM patients with insulin treatment than those with oral hypoglycemic agents treatment.

## Data Availability

All data and material are available by contacting wr1582@163.com

## Abbreviations

DM: Diabetes mellitus
CABG: Coronary artery bypass grafting
AKI: Acute kidney injury
AKIN: Acute kidney injury network
eGFR: Estimated glomerular filtration rate
CPB: Cardiopulmonary bypass
BMI: Body mass index
COPD: Chronic obstructive pulmonary disease
PVD: Peripheral vascular disease
CVA: Cerebro-vascular accident
MI: Myocardial infarction
PCI: Percutaneous coronary intervention
LVEF: Left ventricular ejection fraction
LIMA: Left internal mammary artery
CKD: Chronic kidney disease
